# HTA submission strategies and their associations with rollout times and type of HTA recommendation in Australia and Canada

**DOI:** 10.1017/S0266462326103511

**Published:** 2026-02-09

**Authors:** Belen Sola-Barrado, Ting Wang, Neil McAuslane

**Affiliations:** Centre for Innovation in Regulatory Science (CIRS), London, UK

**Keywords:** health technology assessment (HTA), parallel submissions, Australia, Canada, regulatory HTA alignment

## Abstract

**Objectives:**

Australia and Canada have parallel submission processes allowing companies to submit dossiers to the respective health technology assessment (HTA) body before marketing authorization is issued, aiming to provide more timely access to drugs. This study investigated the associations of submission strategies with new active substance (NAS) rollout times and HTA recommendations.

**Methods:**

This retrospective observational study analyzed HTA appraisals by the Pharmaceutical Benefits Advisory Committee (PBAC) and Canada’s Drug Agency (CDA-AMC) for NASs that received their first HTA recommendation between 2019 and 2023. Regulatory and HTA dates were sourced from public records. We implemented logistic regression to examine associations of HTA recommendation (optimal vs non-optimal). Linear regression was used to test associations of rollout time. Models were adjusted for submission sequence, country, therapeutic area, expedited review, conditional review, top R&D spenders, and year of HTA recommendation.

**Results:**

229 HTA appraisals (126/229 parallel) were included. Parallel submissions were associated with a 14.0-month shorter rollout time compared to sequential submissions (*p* < 0.001). Rollout times in Canada were 6.0 months longer than those in Australia. Parallel submissions were associated with higher odds of receiving an optimal recommendation compared to sequential submissions (OR: 2.2; 95 percent CI: 1.2–4.2; *p* = 0.013). The odds of obtaining an optimal first HTA recommendation were higher in Canada than in Australia.

**Conclusions:**

NASs following parallel submission showed faster rollout times than those following traditional sequential submission. Moreover, parallel submissions were associated with higher odds of receiving an optimal recommendation. These findings highlight the value of aligning regulatory and HTA processes.

## Introduction

Timely patient access to new medicines is a critical objective of healthcare systems. However, medicines must undergo multiple post-development processes before reaching patients, extending the time between clinical development and real-world availability. Improving the efficiency and coordination of these processes is therefore essential to ensure that patients benefit from therapeutic innovation as early as possible.

This road to patient access to new medicines typically encompasses several milestones, including (i) obtaining marketing authorization and (ii) receiving a health technology assessment (HTA) recommendation for reimbursement. These milestones are achieved through a regulatory review process, which is designed to ensure that a new medicine meets safety and efficacy standards (1;2), and an HTA process, which informs decision makers responsible for integrating pharmaceuticals into the healthcare system about the value of a health technology (3). The regulatory review process starts first and is followed by the HTA review process (4;5). Importantly, an HTA recommendation cannot be issued until a positive outcome for marketing authorization is confirmed.

Accelerating the time to HTA recommendation is crucial to ensure patients have the fastest possible access to essential and innovative medicines. To this end, a variety of strategies have been adopted by various health technology assessment bodies (HTAb). Some of these focus on providing flexible pathways to make the HTA review process more efficient, such as rapid reviews in Ireland (6) or abbreviated submissions is Scotland (7). Other strategies do not aim directly at reducing the HTA review time, but at allowing the HTA review process to begin earlier. This is the case for the parallel regulatory/HTA review process, which allows the regulatory and HTA review processes to occur concurrently. Typically, health technology developers must wait until the regulatory process is complete before submitting their dossiers to the HTAb, which can be referred to as a sequential submission process. An alternative approach exists in the form of a parallel submission process. This strategy allows companies to submit their dossier to the HTAb for review after submission to the regulatory agency but before regulatory approval is granted. The primary objective of parallel submissions is to expedite access to new medicines by overlapping the regulatory and HTA review processes.

Parallel regulatory/HTA submissions are available in several countries (*e.* *g.,* Australia, Canada, and the Netherlands) (4;5;8). The Pharmaceutical Benefits Advisory Committee (PBAC) in Australia, and Canada’s Drug Agency (CDA-AMC) in Canada, are widely recognized for their implementation of this process (4;5). Both Australia and Canada operate universal healthcare systems with publicly funded drug benefit schemes (9;10). In Australia, since 2011, sponsors have been permitted to submit concurrently to the Therapeutic Goods Administration (TGA) and PBAC (5). In Canada, since 2018, sponsors have been able to file a submission for review by CDA-AMC up to 180 calendar days in advance of the anticipated date of a Notice of Compliance (NOC) or NOC with conditions by Health Canada (HC) (4). Studies have previously indicated that companies make use of these parallel approaches and typically file their submissions around 107 days before obtaining regulatory approval from the TGA, and approximately a month before securing approval in Canada (11).

Earlier evidence has suggested the advantages of parallel regulatory/HTA reviews in expediting the time to HTA recommendation (11–14). However, these analyses primarily employed descriptive statistics and did not control for confounding factors that might impact the observed timelines. Additional findings from a separate investigation in Australia highlighted associations between parallel submissions and shortened time to HTA recommendation for oncology products (15). However, the focus on anti-cancer therapies limits the generalizability of these conclusions to other therapeutic areas. To bridge this gap, our study leverages 5 years’ worth of public domain data on new active substances (NASs) that received an HTA recommendation by PBAC or CDA-AMC to provide a comprehensive understanding of the associations of submission strategies with both time to HTA recommendation and the type of HTA recommendation. These insights aim to support companies’ strategies and provide agencies with an analysis on the utilization of the parallel process, contributing to the optimization of HTA processes and ultimately facilitating faster patient access to essential and innovative therapies.

## Methods

This was a retrospective observational study using public data from all NASs that received their first HTA recommendation from PBAC, Australia, and/or CDA-AMC, Canada, between January 1, 2019 and December 31, 2023 (*n* = 229).

### Data collection

For Australia, the regulatory submission date was collected as the “Evaluation commenced” date reported by the TGA, while the regulatory approval date was the “Registration decision” date, both obtained from the TGA’s website. The HTA recommendation date was recorded as the fifteenth of the month in which the PBAC meeting making the first HTA recommendation occurred, as reported in the Public Summary Document. The HTA submission date was calculated as 17 weeks prior to the PBAC meeting, as the cut-off date for major submissions is generally 17 weeks before the PBAC meeting. The Pharmaceutical Benefits Scheme (PBS) listing date was collected from the Australia’s PBS website. The HTA outcome was categorized as optimal if the PBAC recommendation was either “recommended” or “recommended” + restrictions, and as non-optimal if the PBAC recommendation was “rejected.” For first HTA recommendations where the HTA outcome was non-optimal, the number of cycles until optimal was calculated as the number of Public Summary Documents published until a PBAC recommendation was optimal.

In Canada, the date in which the sponsor filed the initial submission to HC described within the Summary Basis of Decision for the particular NAS was used as the regulatory submission date. The NOC by HC was used as the regulatory approval date. The HTA submission date was defined as the “Submission received by CDA-AMC” date, and the HTA recommendation date corresponded to the “Final recommendation issued to sponsor and drug plans,” both reported on CDA-AMC’s website. The HTA outcome was categorized as optimal if the recommendation type was either “reimburse” or “reimburse with clinical criteria and/or conditions,” and non-optimal if the recommendation was “do not reimburse.”

For both PBAC and CDA-AMC submissions, submission sequences were classified as sequential if the HTA submission date occurred after the regulatory approval date, and parallel otherwise.

The World Health Organization’s Anatomical Therapeutic Chemical classification was used to determine the therapeutic area of each NAS as follows: Antineoplastic and immunomodulating agents: L (termed Oncology in this study), Nervous system: N, Alimentary and metabolism: A, and Blood and blood-forming organs: B. Expedited review was categorized as Expedited if it was a Priority Review by the TGA in Australia or by HC in Canada. All products assessed through pathways outside this Priority Review definition were classified within Expedited review as non-expedited. Conditional review was defined as Conditional if it was TGA Provisional Approval in Australia and as HC Notice of Compliance with Conditions (NOC/c) in Canada. Top R&D spenders were defined as pharmaceutical companies with R&D expenditures exceeding 3 billion USD in 2020 (16).

### Statistical analysis

The primary outcome of interest was rollout time (in months), defined as the time from submission to the regulatory agency to the date of the first HTA recommendation at the target jurisdiction, which includes both agency and company time. The first HTA recommendation type (coded as “1” for “optimal” and “0” for “non-optimal”) was used as a secondary outcome of interest. We implemented linear regression for our primary outcome of interest and logistic regression for the secondary outcome. The models adjusted for submission sequence (parallel or sequential), country (Australia and Canada), therapeutic area (oncology and non-oncology), expedited review (expedited and non-expedited), conditional review (conditional and non-conditional), top R&D spenders (top and non-top), and year of HTA recommendation (2019, 2020, 2021, 2022, and 2023).

## Results

A total of 229 HTA appraisals of NASs were identified between 2019 and 2023 (95/229 published by PBAC, Australia, and 134/229 by CDA-AMC, Canada). Table 1 shows the characteristics of the appraisals. Among these, 57.9 percent (55/95) of the HTA appraisals in Australia and 53.0 percent (71/134) in Canada were conducted through a parallel regulatory/HTA process. Parallel submissions in Australia resulted in optimal recommendations in 49.1 percent (27/55) of cases, while sequential submissions yielded optimal recommendations in 12.5 percent (5/40) of cases. In Canada, 76.1 percent (54/71) of parallel submissions received an optimal recommendation, and 74.6 percent (47/63) of sequential submissions obtained an optimal recommendation.

**Table 1.** Characteristics of HTA appraisals published in Australia (PBAC) and Canada (CDA-AMC) between 2019 and 2023

	Australia (*n* = 95)		Canada (*n* = 134)	
Characteristic	Sequential *N* = 40	Parallel *N* = 55	Sequential *N* = 63	Parallel *N* = 71
HTA outcome^a^				
Non-optimal (0)	35 (87.5%)	28 (50.9%)	16 (25.4%)	17 (23.9%)
Optimal (1)	5 (12.5%)	27 (49.1%)	47 (74.6%)	54 (76.1%)
Year of first HTA recommendation				
2019	4 (10.0%)	11 (20.0%)	10 (15.9%)	16 (22.5%)
2020	8 (20.0%)	8 (14.5%)	8 (12.7%)	11 (15.5%)
2021	10 (25.0%)	12 (21.8%)	16 (25.4%)	10 (14.1%)
2022	8 (20.0%)	19 (34.5%)	18 (28.6%)	20 (28.2%)
2023	10 (25.0%)	5 (9.1%)	11 (17.5%)	14 (19.7%)
Therapeutic area				
Alimentary and metabolism	3 (7.5%)	3 (5.5%)	4 (6.3%)	5 (7.0%)
Blood and blood-forming organs	2 (5.0%)	1 (1.8%)	4 (6.3%)	2 (2.8%)
Nervous system	7 (17.5%)	8 (14.5%)	10 (15.9%)	6 (8.5%)
Oncology	18 (45.0%)	28 (50.9%)	33 (52.4%)	33 (46.5%)
Other	10 (25.0%)	15 (27.3%)	12 (19.0%)	25 (35.2%)
Expedited review				
Non-expedited	34 (85.0%)	49 (89.1%)	44 (69.8%)	53 (74.6%)
Expedited	6 (15.0%)	6 (10.9%)	19 (30.2%)	18 (25.4%)
Conditional review				
Conditional	4 (10.0%)	9 (16.4%)	13 (20.6%)	8 (11.3%)
Non-conditional	36 (90.0%)	46 (83.6%)	50 (79.4%)	63 (88.7%)
Top R&D spenders internationally				
Top	17 (42.5%)	28 (50.9%)	24 (38.1%)	32 (45.1%)
Non-Top	23 (57.5%)	27 (49.1%)	39 (61.9%)	39 (54.9%)
Rollout time (months)^b^	20.7 (16.0, 26.1)	11.5 (8.5, 13.4)	24.8 (21.2, 33.8)	14.8 (12.8, 18.4)
HTA review time (months)^b^	3.9 (3.9, 3.9)	3.9 (3.9, 3.9)	7.7 (6.7, 9.7)	8.3 (7.2, 10.1)
Regulatory approval to HTA submission time (months)^b^	5.3 (2.1, 8.9)	−3.9 (−6.3, −2.4)	5.9 (2.7, 12.9)	−4.4 (−5.5, −2.8)

a HTA outcome: 0 = “non-optimal,” includes negative HTA recommendations, 1 = “optimal,” includes both positive recommendations and positive with restrictions recommendations.

b Time is displayed as median (interquartile range).

From 2019 to 2022, the parallel review process was the most common submission strategy in Australia compared with sequential review (Supplementary Figure 1). In 2023, there was a decrease in parallel submissions compared with sequential. The percentage of parallel submissions in Canada was generally higher than sequential for all years except in 2021, where sequential submissions presented a higher proportion.

### HTA submission strategy and rollout time

**Figure 1.**
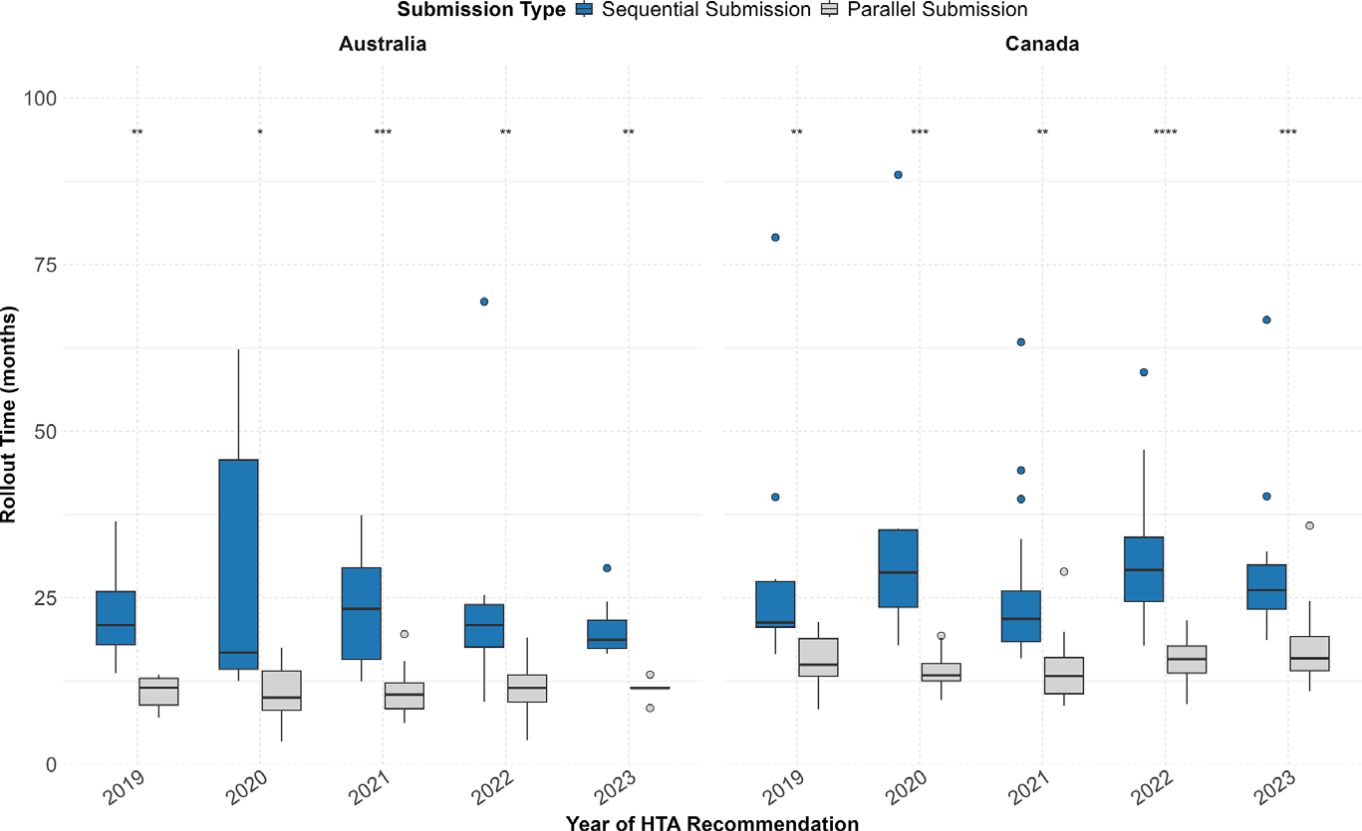
Rollout time of NASs that received their first HTA recommendation between 2019 and 2023 by submission sequence (parallel vs sequential) in Australia and Canada. Australia (sequential: *n* = 40/95 [42.1 percent]; parallel: *n* = 55/95 [57.9 percent]); Canada (sequential: *n* = 63/134 [47.0 percent]; parallel: *n* = 71/134 [53.0 percent]). Statistical significance between submission types (parallel vs sequential) is indicated for each year and country: ****p < 0.0001, ***p < 0.001, **p < 0.01, *p < 0.05.

Figure 1 depicts the rollout time (in months) of NASs that received a first HTA recommendation in Australia and Canada between 2019 and 2023, comparing NASs that underwent a parallel submission process with those that followed sequential submission. Comparing by year of HTA recommendation, NASs that followed a parallel submission strategy presented a significantly shorter median time to HTA recommendation compared with sequential submissions in both countries (Figure 1). Median values are available in Table 1 of the Supplementary Information.

Parallel submissions were associated with a 14.0-month shorter rollout time compared with sequential submissions (*p* < 0.001) (Table 2). Additionally, rollout times to HTA recommendation in Canada were 6.0 months longer than those in Australia after controlling for other factors (*p* < 0.001). Expedited review processes were associated with a 5.5 months shorter rollout time when compared with non-expedited review processes (*p* < 0.001). Non-conditional approvals were associated with a 5.7-month longer rollout time compared to conditionals (*p* = 0.005). Therapeutic areas, applications from top R&D spenders or the year of HTA recommendation did not show a significant difference in rollout time.

**Table 2.** Crude and adjusted estimates for associations with rollout time

	Descriptive		Univariable			Multivariable		
Characteristic	*N* = 229^a^	Mean rollout time months (SD)	*β*	95% CI	*p*-value	*β*	95% CI	*p*-value
Submission sequence								
Sequential	103.0 (45.0%)	27.7 (14.5)	–	—		–	—	
Parallel	126.0 (55.0%)	13.7 (4.7)	−14.0	−17.0, −11.0	**<0.001**	−14.0	−17.0, −12.0	**<0.001**
Country								
Australia	95.0 (41.5%)	16.6 (11.2)	–	—		–	—	
Canada	134.0 (58.5%)	22.4 (12.7)	5.8	2.6, 9.0	**<0.001**	6.0	3.4, 8.7	**<0.001**
Therapeutic area								
Non-oncology	117.0 (51.1%)	21.3 (14.2)	–	—		–	—	
Oncology	112.0 (48.9%)	18.6 (10.1)	−2.6	−5.9, 0.6	0.11	−1.9	−4.6, 0.8	0.2
Expedited review								
Non-expedited	180.0 (78.6%)	20.5 (12.5)	–	—		–	—	
Expedited	49.0 (21.4%)	18.1 (12.4)	−2.4	−6.3, 1.6	0.2	−5.5	−8.7, −2.2	**<0.001**
Conditional review								
Conditional	34.0 (14.8%)	16.4 (5.8)	–	—		–	—	
Non-conditional	195.0 (85.2%)	20.6 (13.2)	4.2	−0.4, 8.7	0.070	5.7	1.8, 9.7	**0.005**
Top R&D spenders								
Top	101.0 (44.1%)	18.5 (10.7)	–	—		–	—	
Non-top	128.0 (55.9%)	21.1 (13.6)	2.6	−0.7, 5.8	0.12	0.6	−2.2, 3.3	0.7
Year HTA recommendation								
2019	41.0 (17.9%)	18.4 (12.0)	–	—		–	—	
2020	35.0 (15.3%)	21.7 (17.7)	3.3	−2.4, 9.0	0.3	1.8	−2.6, 6.2	0.4
2021	48.0 (21.0%)	19.4 (10.9)	1.0	−4.2, 6.3	0.7	−1.4	−5.5, 2.7	0.5
2022	65.0 (28.4%)	19.9 (11.9)	1.5	−3.5, 6.4	0.6	0.8	−3.0, 4.7	0.7
2023	40.0 (17.5%)	20.9 (10.2)	2.5	−3.0, 8.0	0.4	−0.6	−4.8, 3.7	0.8

*Note:* The “Multivariable” column shows mutually adjusted estimates adjusting for submission strategy, country, therapeutic area, expedited review, conditional review, top R&D spender, and year of HTA recommendation. The “Univariable” column shows unadjusted estimates. Significant *p* values are highlighted in bold.

a n (%).

CI, Confidence interval.

The median HTA review times were also studied, and no significant differences were found between the HTA review times of parallel process compared with sequential for all the years studied (see Supplementary Information – Submission type and HTA review time). No overlap between regulatory review time and HTA review time was observed for the sequential processes, as expected. The median overlap durations for parallel processes varied across years and between Australia and Canada. In Australia, the median overlaps were 3.9 months (*n* = 11) in 2019, 4.9 months (*n* = 8) in 2020, 6.0 months (*n* = 12) in 2021, 3.4 months (*n* = 19) in 2022, and 5.1 months (*n* = 5) in 2023. In Canada, the median overlaps were 3.5 months (*n* = 16) in 2019, 5.5 months (*n* = 11) in 2020, 4.8 months (*n* = 10) in 2021, 4.6 months (*n* = 20) in 2022, and 3.5 months (*n* = 14) in 2023.

### HTA submission strategy and the type of HTA recommendation

Table 3 summarizes the characteristics of optimal and non-optimal HTA recommendations included in our analysis, along with crude and adjusted odds ratios (OR) for optimal versus non-optimal recommendations (where an OR greater than 1.0 implies greater odds of obtaining an optimal recommendation). In the text, we refer to the adjusted OR from the multivariable linear regression model unless otherwise stated.

**Table 3.** Crude and adjusted odds ratios (OR) for associations with the type of HTA recommendation

	Outcome		Univariable			Multivariable		
Characteristic	Non-optimal *N* = 96^a^	Optimal *N* = 133^a^	OR	95% CI	*p*-value	OR	95% CI	*p*-value
Submission sequence								
Sequential	51.0 (49.5%)	52.0 (50.5%)	–	—		–	—	
Parallel	45.0 (35.7%)	81.0 (64.3%)	1.8	1.0, 3.0	**0.036**	2.2	1.2, 4.2	**0.013**
Country								
Australia	63.0 (66.3%)	32.0 (33.7%)	–	—		–	—	
Canada	33.0 (24.6%)	101.0 (75.4%)	6.0	3.4, 11.0	**<0.001**	7.4	4.0, 14.4	**<0.001**
Therapeutic area								
Non-oncology	46.0 (39.3%)	71.0 (60.7%)	–	—		–	—	
Oncology	50.0 (44.6%)	62.0 (55.4%)	0.8	0.5, 1.4	0.4	0.8	0.4, 1.6	0.6
Expedited review								
Non-expedited	81.0 (45.0%)	99.0 (55.0%)	–	—		–	—	
Expedited	15.0 (30.6%)	34.0 (69.4%)	1.9	1.0, 3.7	0.073	1.3	0.6, 3.0	0.5
Conditional review								
Conditional	19.0 (55.9%)	15.0 (44.1%)	–	—		–	—	
Non-conditional	77.0 (39.5%)	118.0 (60.5%)	1.9	0.9, 4.1	0.077	2.5	1.0, 6.2	0.052
Top R&D spenders internationally								
Top	42.0 (41.6%)	59.0 (58.4%)	–	—		–	—	
Non-top	54.0 (42.2%)	74.0 (57.8%)	1.0	0.6, 1.7	>0.9	0.7	0.4, 1.3	0.3
Year HTA recommendation								
2019	15.0 (36.6%)	26.0 (63.4%)	–	—		–	—	
2020	13.0 (37.1%)	22.0 (62.9%)	1.0	0.38, 2.51	>0.9	1.41	0.5, 4.2	0.5
2021	20.0 (41.7%)	28.0 (58.3%)	0.8	0.34, 1.90	0.6	1.01	0.4, 2.7	>0.9
2022	29.0 (44.6%)	36.0 (55.4%)	0.7	0.32, 1.59	0.4	0.86	0.3, 2.1	0.7
2023	19.0 (47.5%)	21.0 (52.5%)	0.6	0.26, 1.54	0.3	0.62	0.2, 1.7	0.4

*Note*: The “Multivariable” column shows mutually adjusted OR for all variables show in the table. The “Univariable” column shows unadjusted crude ORs.

a n (%).

CI, confidence interval; OR, odds ratio.

The submission strategy was significantly associated with the type of HTA recommendation. Specifically, parallel submissions were associated with higher odds of receiving an optimal recommendation compared with sequential submissions (OR: 2.2; 95 percent CI: 1.2, 4.2; *p* = 0.013). Additionally, the odds of obtaining an optimal first HTA recommendation were higher in Canada than in Australia (OR: 7.4; 95 percent CI: 4.0, 14.4; *p* < 0.001).

Oncology applications, when compared with non-oncology, did not show a significant difference in the odds of receiving an optimal recommendation. Similarly, no differences were observed in the type of recommendation by drug class or other characteristics.

### Focus on Australia – Submission strategy and listing

A medicine can only be reimbursed in Australia (listed on the PBS) after an optimal recommendation from PBAC. When the PBAC’s first HTA recommendation is negative, companies can resubmit an application with an improved dossier. Consequently, several review cycles may occur until an optimal recommendation is achieved. 12.5 percent (5/40) of sequential submissions received an optimal HTA recommendation in their first recommendation from PBAC, compared to 49.1 percent (27/55) of parallel submissions that received an optimal recommendation in their first recommendation.

Of the sequential submissions, 50.0 percent (20/40) needed at least one resubmission (one resubmission or more) to reach an optimal HTA recommendation from PBAC. Of the parallel submissions, 25.5 percent (14/55) needed at least one resubmission to PBAC to reach an optimal recommendation. Finally, 37.5 percent (15/40) and 25.5 percent (14/55) of the sequential and parallel submissions, respectively, still had not received an optimal recommendation by the time our data collection was completed.

**Figure 2.**
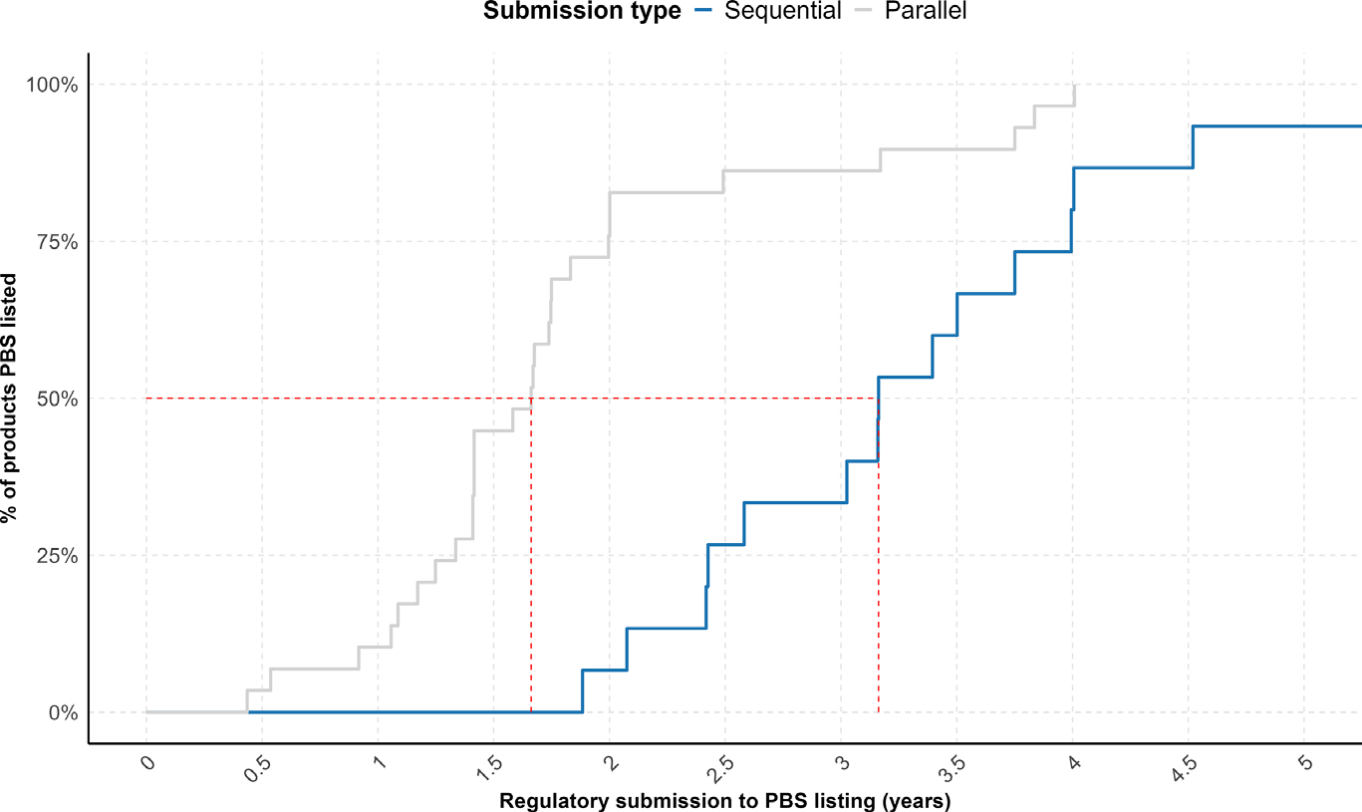
Cumulative plot showing the proportion of NASs following sequential submissions (*n* = 15) (blue) and parallel submissions (*n* = 29) (gray) that received a first HTA recommendation from PBAC between 2019 and 2023 and were subsequently listed on the PBS in Australia over time. The red dashed line indicates the time points (X-axis intercepts) at which 50 percent of products in each cohort achieved PBS listing.

The proportion of listed products varied between the two submission strategies, with 37.5 percent (15/40) of sequential submissions and 52.7 percent (29/55) of parallel submissions achieving listing in the PBS during the time window measured. In addition, a cumulative analysis of time-to-listing for the cohort of listed products, using regulatory submission dates as the starting point, demonstrated that parallel submissions achieved significantly faster PBS listings (*p* = 0.0004) (Figure 2). Overall, the median time from regulatory submission to PBS listing was 3.2 years for sequential submissions and 1.7 years for parallel submissions.

## Discussion

This study shows that between 2019 and 2023, NASs consistently presented a faster median rollout time from regulatory submission to HTA recommendation if they had followed a parallel regulatory/HTA review compared to sequential, both in Australia and Canada. In line with this, we found that the submission strategy was significantly associated with rollout time, with parallel submissions showing an average rollout time of 14 months faster than sequential submissions.

Earlier studies have suggested the advantages of parallel regulatory/HTA reviews in expediting the time to HTA recommendation (11;12;14). However, these investigations relied solely on descriptive statistics. Furthermore, such approaches were limited in their ability to account for potential confounding factors that could influence the timelines under investigation. The present research addresses these limitations by employing regression modeling strategies to examine the relationship between submission strategies and rollout times. By controlling for confounding variables and utilizing regression analysis, this study provides a more comprehensive understanding of the factors potentially influencing the timing of HTA recommendations, such as the submission sequence, the country, the therapeutic area, or the review type.

Another study also provided important insights into the associations of parallel submissions and the time to HTA recommendations for oncology products in Australia (15). In line with our findings, the study found that the parallel process was associated with a reduced lag between regulatory approval and PBAC funding recommendation for oncology products compared with the sequential process. However, the study’s scope was limited to oncology drugs, leaving the effectiveness of parallel processing for non-oncology therapeutics unknown. Our study explores submissions across all therapeutic categories and includes data not only from Australia but also from Canada, where this process is also available. In addition, we showed that NASs following parallel submissions achieved significantly faster PBS listings compared to sequential submissions, effectively presenting faster patient access to new medicines. The latter may have been influenced not only by the faster parallel process, but also by the fact that, in Australia, parallel submissions presented a higher proportion of optimal recommendations compared to sequential submissions.

Our study did not examine resubmission cycles to CDA-AMC in Canada because, unlike in Australia, repeated submission–reassessment cycles are not a routine feature of the Canadian process (4). We also did not examine public reimbursement listing dates in Canada because there is no single national public drug plan; listings are determined separately by each provincial and territorial drug program (17).

Our analysis showed that expedited review processes were associated with a shorter rollout time when compared with non-expedited review processes. This was expected as expedited reviews present different timeline targets for both the TGA and HC: within 150 working days for a TGA Priority Review (vs ~255 working days for standard reviews) and within 180 calendar days for a HC Priority Review (vs ~300 calendar days for standard reviews). The results indicated that conditional approvals were associated with a faster rollout time compared to non-conditional. This result may have been influenced by the faster review target for conditional approvals compared to standard by both TGA (220 working days vs 255, respectively) and HC (200 calendar days vs 300, respectively) (18;19). In addition, we did not observe any association between conditional approvals and the type of HTA recommendation.

The median HTA review times were also studied, and we found no significant difference between the HTA review times of parallel process compared to sequential for all the years studied. This finding is expected in Australia as the time required for PBAC to reach a decision from the time of submission is fixed. The PBAC operates on a 17-week cycle, with submission windows available three times a year – in March, July, and November – regardless of whether the process is parallel or sequential (20;21). Similarly in Canada, CDA-AMC indicates that a typical timeline for an HTA review completion is ≤180 days, also independently of whether this is related to a parallel or sequential review (4). Although HTA review timelines are shown to be independent of the submission strategy, our study indicates that overlapping the regulatory and HTA review processes significantly reduced the overall rollout time.

We found that the submission strategy was significantly associated with the type of HTA recommendation, with parallel submissions being associated with higher odds of receiving an optimal recommendation compared to sequential submissions. A potential rationale for this result could be that companies that address both regulatory and HTA evidence needs during the development phase can be ready to meet the requirements of these two bodies in parallel. On the other hand, companies prioritizing regulatory evidentiary requirements during the development phase may need more preparatory time before submitting to an HTA body, having to submit in a sequential manner, and may find meeting the HTA evidentiary requirements more challenging since the development phase is completed. This hypothesis is in line with a previously published study that reported that it was more common that products submitted in parallel in Australia provided the same pivotal evidence to the regulator and HTA agency, differently from sequential submissions (15).

Our observation reinforces the importance of companies integrating both regulatory and HTA considerations early in the drug development process to streamline submission processes and ultimately increase the chances of successful market access. A number of collaborative strategies are already in place to support companies harmonizing their evidence generation, ensuring it addresses both regulatory and HTA needs. For instance, the new EU HTA Regulation allows joint scientific consultations on medicinal products to take place in parallel with scientific advice from the European Medicines Agency (EMA). This parallel scientific advice is “carried out with a view to ensuring that the generation of evidence fulfils the needs of the respective frameworks, while preserving the separation of their respective remits” (22). In another example, the UK’s Innovative Licensing and Access Pathway (ILAP) allows the developer to engage with both the regulatory and HTA bodies from the early stages of clinical development in order to understand future evidential requirements (23). In 2019, HC and CDA-AMC also launched an initiative to provide early parallel scientific advice (24). This approach enabled both organizations to work together and exchange viewpoints while independently developing their respective recommendations on a sponsor’s drug development strategy.

Additionally, our study found that the odds of obtaining an optimal first HTA recommendation were higher in Canada than in Australia. The reason for this may lie in the particularities of the HTA systems of both countries. As reported in our results, PBAC normally presents a faster median rollout time to the first recommendation compared with CDA-AMC. However, 66.3 percent of PBAC’s first recommendations included in this study were negative, in contrast with only 24.6 percent of first recommendations in Canada. After a first negative recommendation by PBAC, a product can go through a number of re-submission cycles until an optimal recommendation is reached.

The Australian government has previously raised concerns about having fast first reimbursement decisions but two-thirds of these decisions being negative (25), since this means that an important percentage of new therapies in Australia are currently having to go through multiple resubmission cycles to PBAC before obtaining an optimal reimbursement recommendation, which can subsequently result in lengthy delays to PBS listing. To address this issue, the Health Technology Assessment Policy and Methods Review (25), published by the Australian Government in March 2024, proposed a number of suggestions, such as an alternative listing pathway that would be initiated when PBAC considers that a medicine has unproven cost-effectiveness but has clear added therapeutic value. In these cases, instead of a rejection followed by a formal resubmission, the therapy would be temporarily listed while the sponsor and the Department of Health negotiate on price outside the 17-week assessment cycle before a new resubmission to PBAC for a decision.

In terms of submission trends, our study identified that 57.9 percent of the HTA appraisals in Australia and 53.0 percent in Canada were conducted through a parallel regulatory/HTA process. These proportions indicate that, overall, the use of both submission strategies was fairly distributed in both studied countries. However, breaking down by year of HTA recommendation, we observed that from 2019 to 2022 the parallel review process was the most common submission pathway in Australia, but in 2023, sequential submission was the most utilized submission strategy. It is currently unclear whether this shift reflects an emerging trend or a one-off occurrence, and further monitoring will be needed to determine whether underlying drivers may be influencing a longer-term change in submission behaviors.

Our analysis also included as one of the studied variables whether a company’s R&D spend exceeded 3 billion USD (referred to as top companies) (16). We observed that both top and non-top companies appear to utilize parallel submissions in a similar proportion in Australia and Canada, indicating that the benefits of parallel submissions are recognized across the pharmaceutical industry, regardless of R&D investment levels. This highlights that a wide range of companies are benefitting from this strategy, potentially fostering innovation and competition in the pharmaceutical industry.

This study has several strengths. Firstly, it is the first study that has investigated associations of companies’ submission strategy with both the time to HTA recommendation and the type of HTA outcome in a cohort of HTA appraisals from two major jurisdictions, Australia and Canada, across all therapeutic areas. Secondly, this is the first study demonstrating that parallel submissions in Australia achieved significantly faster PBS listings compared to sequential submissions. There are some limitations to our study. We focused our analysis on NASs, therefore any MLE applications were excluded. Applications related to vaccines were also excluded. Future studies could explore additional jurisdictions where the parallel/HTA process is available, such the National Health Care Institute (ZIN) in the Netherlands, which could provide additional insights.

## Conclusion

This study found that parallel regulatory/HTA submissions are associated with shorter rollout times compared to sequential submissions in Australia and Canada. In Australia, parallel submissions also presented significantly faster listings on the PBS. These findings highlight the potential of parallel submissions to expedite patient access to new medicines. Furthermore, submission strategy was significantly associated with HTA outcomes, with parallel submissions associated with higher odds of receiving an optimal recommendation compared to sequential submissions. However, the underlying drivers of the latter association remain unclear and should be explored further. Taken together, these results indicate that, within Australia and Canada, parallel submissions are fit for purpose and should be strongly considered by companies seeking to improve the efficiency of their market entry strategies.

## Supporting information

10.1017/S0266462326103511.sm001 Sola-Barrado et al. supplementary material Sola-Barrado et al. supplementary material

## Supplementary material

The supplementary material for this article can be found at http://doi.org/10.1017/S0266462326103511.
