# Supplementary material for: HTA submission strategies and their associations with rollout times and type of HTA recommendation in Australia and Canada
Source: Int J Technol Assess Health Care. 2026 Feb 9;42(1):e20. doi: 10.1017/S0266462326103511 (PMC12964150; doi:10.1017/S0266462326103511)
Supplement: Sola-Barrado et al. supplementary material [file S0266462326103511sup001.docx]

**Supplementary Information**

**Supplementary Figure 1.** Percentage of HTA recommendations published between 2019 and 2023 submitted via parallel (blue) and sequential (grey) regulatory/HTA processes in Australia and Canada.


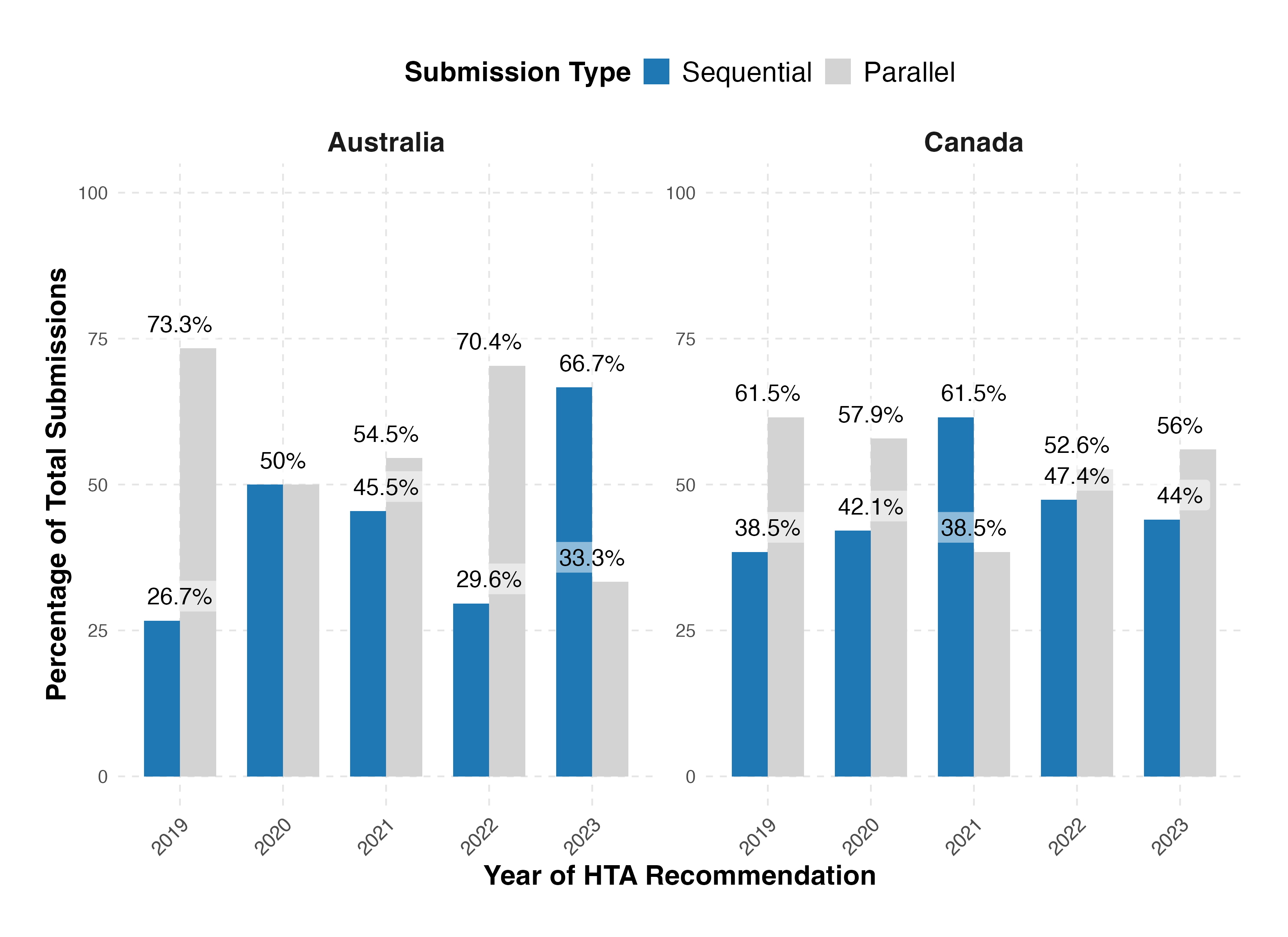


## ***HTA submission strategy and the rollout time***

**Supplementary Table 1**. Median rollout time (months) by country, year of HTA recommendation, and submission strategy (parallel vs sequential).

| Country | Year of HTA Recommendation | p-value^1^ | Median rollout time (months) for Sequential submissions | Median rollout time (months) for Parallel submissions |
| --- | --- | --- | --- | --- |
| Australia | 2019 | 0.0040 | 20.9 | 11.5 |
| Australia | 2020 | 0.0180 | 16.8 | 10.0 |
| Australia | 2021 | 0.0008 | 23.3 | 10.4 |
| Australia | 2022 | 0.0017 | 20.9 | 11.5 |
| Australia | 2023 | 0.0022 | 18.7 | 11.4 |
| Canada | 2019 | 0.0013 | 21.3 | 15.0 |
| Canada | 2020 | 0.0005 | 28.8 | 13.4 |
| Canada | 2021 | 0.0013 | 21.8 | 13.3 |
| Canada | 2022 | 0.0000 | 29.2 | 15.8 |
| Canada | 2023 | 0.0008 | 26.2 | 15.9 |

^1^p-values were calculated with Kruskal–Wallis test.

***Submission type and HTA review time***

The median HTA review times were also studied, and no significant differences were found between the HTA review times of parallel process compared with sequential for all the years studied. In Australia, the median review time was 3.9 months for both parallel and sequential submissions from 2019 to 2023 (except parallel submissions in 2020, which presented a median time of 4.4 months). In Canada, the median review times showed some variability, though values remained relatively similar across both submission processes. In 2019, parallel submissions had a median review time of 8.3 months, compared to 7.1 months for sequential submissions (p = 0.0651). In 2020, parallel submissions had a median review time of 7.8 months, while sequential submissions were slightly higher at 8.5 months (p = 0.9342). In 2021, the median review time for parallel submissions increased to 9.1 months, compared to 7.8 months for sequential submissions (p = 0.2462). By 2022, parallel submissions had a median review time of 9.1 months, while sequential submissions were lower at 7.5 months (p = 0.3346). In 2023, parallel submissions had a median review time of 8.2 months, whereas sequential submissions increased to 9.0 months (p = 0.8695).
